# Role of ZNF652 in Regulating Hepatocellular Carcinoma Cell Proliferation and Apoptosis via the circRHOT1/SLC38A6 Axis

**DOI:** 10.1002/kjm2.70129

**Published:** 2025-11-28

**Authors:** Kang Chen, Fei‐Hu Sun, Chen Fan, Wei Ding, Hao‐Huan Tang, Lei Sun, Wei‐Tao Wang, Wei‐Dong Wang

**Affiliations:** ^1^ Department of Interventional Radiology The Affiliated Wuxi People's Hospital of Nanjing Medical University, Wuxi People's Hospital, Wuxi Medical Center, Nanjing Medical University Wuxi People's Republic of China

**Keywords:** circRHOT1, hepatocellular carcinoma, KAT5, SLC38A6, ZNF652

## Abstract

Hepatocellular carcinoma (HCC) is a heterogeneous malignancy characterized by high mortality rates. This article presents a discussion of the role of ZNF652 in HCC cell proliferation and apoptosis, thereby identifying a new target for HCC treatment. The expression levels of ZNF652, circRHOT1, and SLC38A6 in HCC and healthy cells were analyzed. Cell proliferation and apoptosis were subsequently validated. The binding relationships between ZNF652 and the circRHOT1 promoter and between circRHOT1 and KAT5 were validated. The recruitment of KAT5 and H3K27ac to the SLC38A6 promoter was assessed via ChIP. Combined experiments were carried out to verify the role of the circRHOT1/SLC38A6 pathway in HCC cell proliferation and apoptosis. ZNF652, circRHOT1, and SLC38A6 were upregulated in HCC cells. ZNF652 silencing inhibited HCC cell proliferation but promoted apoptosis. Mechanistically, ZNF652 increased circRHOT1 expression at the transcriptional level and recruited KAT5 to the SLC38A6 promoter to increase H3K27ac enrichment and activate SLC38A6 expression. Combined experiments revealed that overexpression of circRHOT1 or SLC38A6 could alleviate the effects of ZNF652 silencing on HCC cell proliferation and apoptosis. In conclusion, ZNF652 transcriptionally activated circRHOT1 expression, recruited KAT5 to the SLC38A6 promoter, increased H3K27ac enrichment, and activated SLC38A6 expression, thus promoting HCC cell proliferation and inhibiting apoptosis.

## 1 Introduction

Hepatocellular carcinoma (HCC) represents a tremendous challenge for global health because of its high incidence rate and undesirable prognostic outcomes [1]. Limited by its profound heterogeneity and complicated pathogenesis, HCC is difficult to detect and treat and is frequently associated with poor survival outcomes and accelerated metastasis [2]. Worse still, although traditional risk factors, such as hepatitis, liver disorders, alcohol consumption, and excessive exposure to daily toxins, could be controlled within a reasonable range, effective strategies for predicting HCC remain unestablished [1]. Currently, chemotherapy, drug administration, and immunotherapy are considered attractive strategies for preventing HCC, yet treatment is often hindered by side effects, drug resistance, and adverse reactions [3]. Cellular biological activities are pivotal to HCC pathogenesis and progression, as malignant cell proliferation aggravates tumor growth and expansion, whereas apoptosis induces cancer cell dysfunction and loss [4]. In this context, biological targets for HCC cell proliferation and apoptosis are urgently needed.

The zinc finger protein family is a group of crucial DNA‐binding transcription factors that affect cellular proliferation, renewal, senescence, apoptosis, and death [5]. Multiple studies have highlighted the versatile functions of zinc finger proteins in human solid tumors [6, 7]. Zinc finger protein 652 (ZNF652) is strongly expressed in prostate cancer, which has a high incidence [8]. High expression of ZNF652 leads to reduced progression‐free survival and poor prognosis in patients with a high risk of developing breast cancer [9]. Elucidating the binding relationship between ZNF652 and its downstream genes represents a significant breakthrough in advancing cancer therapy [10]. Circular RNAs (circRNAs) refer to a series of conserved single‐chained RNAs that form a closed‐loop structure and are involved in cancer pathogenesis, metastasis, and treatment [11]. Functionally, the circular RNA Rho GTPase 1 (circRHOT1) can be modulated by ZNF652 to promote malignant bladder cancer cell proliferation and metastasis while impairing cell sensitivity to effective treatment [12]. High expression of circRHOT1 is correlated with poor prognosis and increased cancer cell growth, mobility, and proliferation [13]. circRHOT1 accumulates lysine acetyltransferase 5 (KAT5) to increase histone H3 lysine 27 acetylation (H3K27ac) of its downstream gene to promote lung cancer pathogenesis and proliferation while downregulating apoptosis [14]. H3K27ac depletion contributes to a reduction in the initiation and progression of HCC [15]. Notably, enrichment of H3K27ac on solute carrier family 38 member 6 (SLC38A6) in HCC results in reduced cell death and increased cell proliferation, accumulation, and invasion [16]. Overall, we hypothesize that ZNF652 might be a detrimental factor in HCC treatment and perform functional assays to determine the concrete function of ZNF652 in HCC cell proliferation and apoptosis via manipulation of the circRHOT1/SLC38A6 axis, thus extending the possibility of HCC suppression.

## 2 Methods

### 2.1 Cell Culture

HCC cell lines (Huh‐7, MHCC97‐H, HCCLM3, and HCC36) (all from Type Culture Collection of the Chinese Academy of Sciences, Shanghai, China) and transformed human liver epithelial‐2 (THLE‐2) cells (American Type Culture Collection, ATCC, Manassas, VA, USA) were cultured in RPMI‐1640 medium (Gibco, Grand Island, NY, USA) supplemented with 10% fetal bovine serum (Gibco) and 1% penicillin–streptomycin (Gibco) in an incubator with 5% CO_2_ at 37°C.

### 2.2 Cell Treatment

Small interfering RNAs (siRNAs) targeting the ZNF652 and pcDNA3.1 vectors of circRHOT1 and SLC38A6 (GenePharma, Shanghai, China) were transfected into cells according to the instructions of Lipofectamine 3000 transfection reagent (Invitrogen Inc., Carlsbad, CA, USA).

### 2.3 Cell Counting Kit‐8 (CCK‐8) Method

Cell proliferation was assessed using a CCK‐8 kit (Dojindo Molecular Technologies, Kumamoto, Japan). In brief, cells (3 × 10^3^/well) in the logarithmic phase were inoculated into 96‐well plates for 24, 48, or 72 h, after which they were coupled by cultivation with CCK‐8 solution (10 μL/well) for 1 h. The optical density at a wavelength of 450 nm was determined using a microplate reader.

### 2.4 Colony Formation Assay

Cells (1 × 10^3^/well) were seeded into incubator (6 cm) plates for 10 days, after which the colonies were fastened with paraformaldehyde for 15 min and stained with 0.5% crystal violet (Solarbio Science & Technology Co. Ltd., Beijing, China) for 30 min. Afterwards, the number of colonies was counted, and the number of colonies over 50 was recorded.

### 2.5 Flow Cytometry

Apoptosis was measured with an Annexin V‐fluorescein isothiocyanate (FITC)/propidium iodide (PI) kit (Becton, Dickinson and Company, Franklin Lakes, NJ, USA). Cells were then harvested using ethylene diamine tetraacetic acid‐free trypsin, cleaned twice with cold phosphate‐buffered saline, rinsed once with cold binding buffer solution, suspended in 200 μL of cold binding buffer solution, and cultured in Annexin V‐FITC staining solution and PI staining solution for 15 min. In addition, the samples were assessed on a flow cytometer (Beckman Coulter, Brea, CA, USA).

### 2.6 RNase R and Actinomycin D (ACT‐D) Treatment

To analyze the stability between the circular isoform and linear isoform of RHOT1, cells were exposed to ACT‐D (5 μg/mL; Biosharp, Beijing, China) for 8, 16, or 24 h to inhibit transcription. Furthermore, to digest RNase R, 2 μg of RNA was incubated with 8 U of RNase R (Sigma–Aldrich, St. Louis, MO, USA) at 37°C for 30 min, and the transcription level was detected via reverse transcription quantitative polymerase chain reaction (RT–qPCR).

### 2.7 Chromatin Immunoprecipitation (ChIP)

ChIP assays were performed using a ChIP assay kit (Invitrogen). Cells were collected, treated, fastened, and lysed to harvest the genome. Chromatin fragments obtained by ultrasound were rotated and cultured with antibodies against ZNF652 (ab126880), KAT5 (ab300521), H3K27ac (ab4729), and immunoglobulin G (IgG, ab37373/ab172730) (all from Abcam Inc., Cambridge, MA, USA) at 4°C overnight, followed by 2 h of incubation with protein A agarose. The precipitated complexes were subsequently rinsed and eluted. DNA binding was evaluated via RT–qPCR.

### 2.8 Database Prediction

The expression of ZNF652 in HCC was predicted through the Starbase database (https://rnasysu.com/encori/panCancer.php) [17], UALCAN database (https://ualcan.path.uab.edu/index.html) [18], and GEPIA database (http://gepia.cancer‐pku.cn/detail.php) [19]. The binding sites between ZNF652 and the circRHOT1 promoter were predicted through the JASPAR database (https://jaspar.elixir.no/) [20].

### 2.9 Dual‐Luciferase Reporter Gene Assay

CircRHOT1 fragments with wild‐type (WT) or mutant (MUT) binding sites were cloned and inserted into the pGL3 vector (Promega, Fitchburg, WI, USA) to construct the luciferase reporter vectors WT‐circRHOT1 and MUT‐circRHOT1, which were then cotransfected into cells with pcDNA3.1 NC or pcDNA3.1 ZNF652. Luciferase activity was determined using a dual‐luciferase reporter gene assay kit (Promega).

### 2.10 RNA Immunoprecipitation (RIP) Assay

The cells were fixed using 1% formaldehyde for 10 min and then lysed using radioimmunoprecipitation assay (RIPA) lysis buffer supplemented with an RNase inhibitor and a protease inhibitor (Sigma–Aldrich). The samples were fractured using ultrasound and centrifuged to collect the supernatant, which was incubated with protein A/G beads (Cell Signaling Technology, Danvers, MA, USA) for 1 h and incubated with anti‐KAT5 antibody (ab300521; Abcam) for 4 h, with IgG (ab172730; Abcam) used as the control. The enrichment of circRHOT1 was examined via RT–qPCR.

### 2.11 RNA Pull‐Down Assay

CircRHOT1 and its antisense protein biotinylated RNA probes were synthesized and collected from Genechem (Shanghai, China). The assay was carried out according to the manufacturer's guidelines. Biotin‐labeled circRHOT1 was cross‐linked with beads and cultured with cell lysis solution. Afterwards, the precipitated complexes were rinsed and heated to collect protein samples for subsequent western blot experiments.

### 2.12 RT–qPCR

Total RNA was isolated from cells with TRIzol reagent (Invitrogen) and quantified using a NanoDrop 2000 (Thermo Fisher Scientific Inc., Waltham, CA, USA). Complementary DNA was synthesized using a PrimeScript RT Reagent Kit (Takara, Shiga, Japan). RT–qPCR was carried out on an Applied Biosystems 7500 system with a SYBR Primer‐Script RT–PCR kit (Takara), with glyceraldehyde‐3‐phosphate dehydrogenase (GAPDH) as the internal reference. The 2^−ΔΔCT^ method was used to measure relative expression [21]. The primers used are listed in Table 1.

**TABLE 1:** Primers sequence of RT‐qPCR.

Name of primer	Sequence (5′‐3′)
ZNF652	F:AGACACCGCAGAACTCACAC
	R:CACCCGAAAGCACTTCTCCT
circRHOT1	F:AAGAACAGACAAAGACAGCAG
	R:TTCTTCTGCCCGGGGAGGAAC
RHOT1	F:GCTGGTGGGAGAACCTAGAG
	R:TGGAACTCTCTCTGGGGTGA
SLC38A6	F:TGTCCAGCAGCCTGAAGAAG
	R:CCAAGGATGCCACTTCCCAT
circRHOT1 promoter	F:CACAGAGCTATTATGTGGAGC
	R:GAATGAAATGTGCGTAATACC
SLC38A6 promoter	F:CCCTTGGCTCATTTCCG
	R:TCCTTCGCCTTCCTTTCC
GAPDH	F:GTCAAGGCTGAGAACGGGAA
	R:TCGCCCCACTTGATTTTGGA

Abbreviations: circRHOT1, circular RNA Rho GTPase 1; F, forward; GAPDH, glyceraldehyde‐3‐phosphate dehydrogenase; R, reverse; RT‐qPCR, reverse transcription‐quantitative polymerase chain reaction; SLC38A6, solute carrier family 38 member 6; ZNF652, zinc finger protein 652.

### 2.13 Western Blot Analysis

Cells were lysed with RIPA buffer solution (Beyotime Biotechnology Co. Ltd., Shanghai, China) to separate total protein, which was detected with a bicinchoninic acid protein assay kit (TIANGEN, Beijing, China). Afterwards, protein was separated via sodium dodecyl sulfate–polyacrylamide gel electrophoresis (Solarbio) and then transferred onto polyvinylidene fluoride membranes (Sigma–Aldrich), which were then sealed using skim milk for 2 h and cultured with antibodies against ZNF652 (1:1000; ab126880), KAT5 (1:1000; ab300521), SLC38A6 (1:1000; ab237767), B‐cell lymphoma‐2 (Bcl‐2, 1:2000; ab182858), Bcl‐2‐associated X (Bax, 1:1000; ab32503), and GAPDH (1:2500; ab9485) (all from Abcam) overnight. The membranes were subsequently incubated with secondary antibody (1:2000; ab97110/ab205718; Abcam) at room temperature for 2 h. An enhanced chemiluminescence assay (Sigma–Aldrich) was used to visualize the signal. Additionally, protein bands were visualized using enhanced chemiluminescence reagent (Vazyme, Nanjing, Jiangsu, China), with GAPDH functioning as the internal reference. Additionally, ImageJ software was used to calculate the gray value intensity of the target protein band.

### 2.14 Statistical Analysis

SPSS 21.0 (IBM SPSS Statistics, Chicago, IL, USA) was used to verify the data, and GraphPad Prism 8.0 software (GraphPad Software Inc., San Diego, CA, USA) was used to generate the graphs. Our data were tested for a normal distribution and homogeneity of variance, and the data are presented as the mean ± standard deviation. Comparisons between two groups were performed with a *t* test, and comparisons among various groups were performed with one‐way or two‐way analysis of variance (ANOVA). Tukey's multiple comparisons test was used for post hoc tests. The *p* value was calculated by a two‐tailed test, with *p* < 0.05 indicating statistical significance.

## 3 Results

### 3.1 ZNF652 Accelerates HCC Cell Proliferation and Represses Apoptosis

**FIGURE 1:**
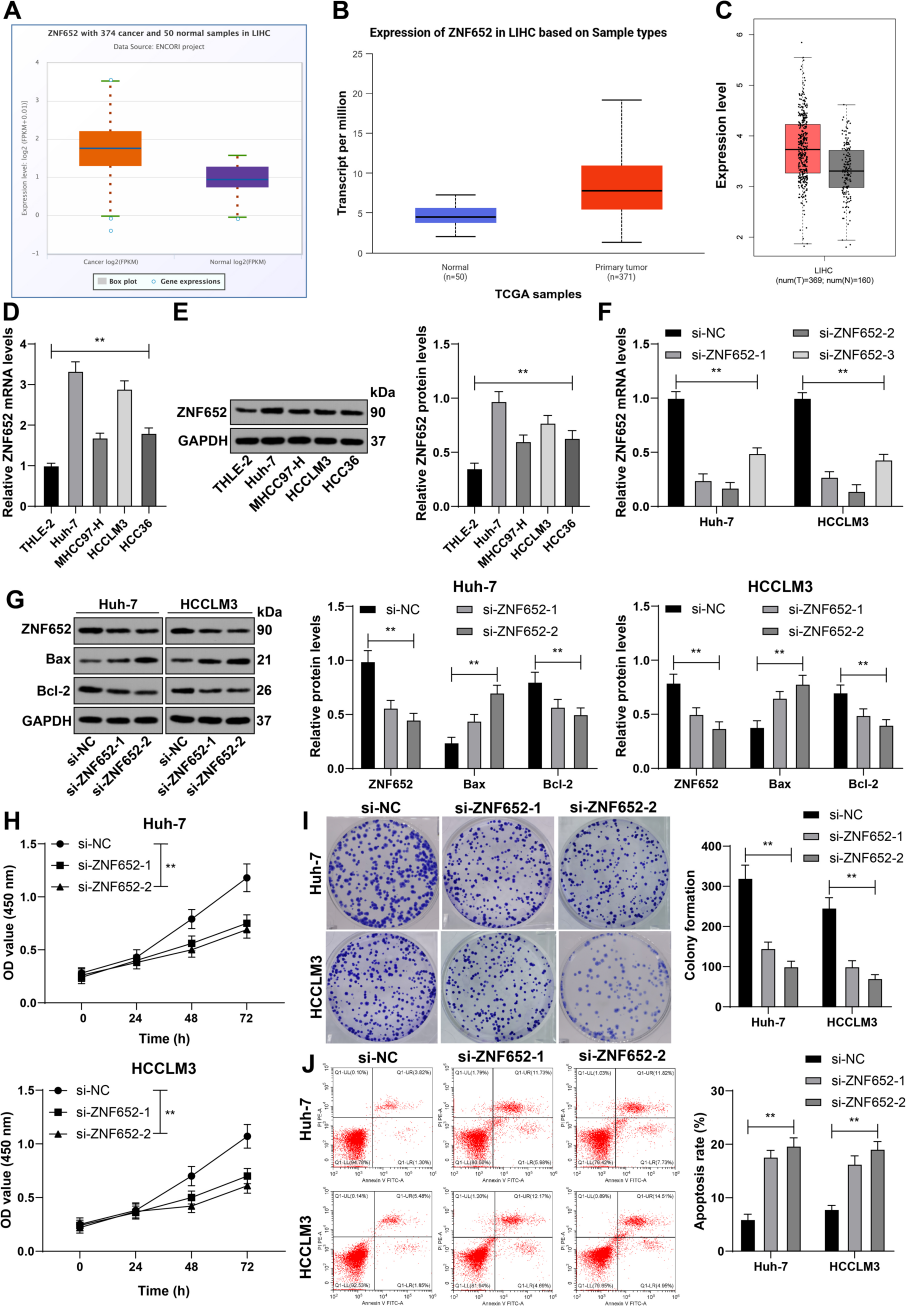
ZNF652 accelerates HCC cell proliferation and represses apoptosis. (A–C) ZNF652 expression in hepatocellular carcinoma was predicted through databases. (D and E) Cellular ZNF652 expression was examined via RT–qPCR (D) and western blot analysis (E), and si‐ZNF652 was transfected into HCC cell lines, with si‐NC as the NC. (F) ZNF652 transfection efficiency was examined via RT–qPCR. (G) The expression of ZNF652, Bax and Bcl‐2 was detected via western blot analysis. (H) Cell proliferation was verified via the CCK‐8 method. (I) The number of cell colonies was evaluated using a colony formation assay. (J) Apoptosis was assessed via flow cytometry. Three independent operations were conducted. The data are presented as the means ± standard deviations. One‐way ANOVA was used to compare the data among multiple groups in Panels D and E, and two‐way ANOVA was used to compare the data among multiple groups in Panels F–J. Tukey's multiple comparisons test was used for post hoc tests. **p < 0.01.

The results from the databases revealed that ZNF652 expression was upregulated in HCC (Figure 1A–C). In this case, cell lines were cultured to detect ZNF652 expression via RT–qPCR and western blot analysis. Compared with that in THLE‐2 cells, the mRNA level of ZNF652 in HCC cell lines was elevated (*p* < 0.01; Figure 1D,E). Subsequently, Huh‐7 and HCCLM3 cell lines with higher ZNF652 expression were chosen for the following procedures, and si‐ZNF652 was transfected into the cells to inactivate ZNF652 expression (*p* < 0.01; Figure 1F,G). si‐ZNF652‐1 and si‐ZNF652‐2, with better transfection efficiency, were chosen for the following procedures. The proliferation viability of the HCC cell lines showed decreased proliferation viability and decreased colony number, as did the number of colonies, upon ZNF652 silencing (*p* < 0.01; Figure 1H,I). Compared with the si‐NC group, the si‐ZNF652 group exhibited enhanced HCC apoptosis, elevated Bax protein expression, and suppressed Bcl‐2 protein expression (*p* < 0.01; Figure 1G,J). Overall, ZNF652 was strongly expressed in the HCC cell lines, whereas ZNF652 silencing inhibited HCC cell proliferation and promoted apoptosis.

### 3.2 ZNF652 Transcriptionally Promotes circRHOT1 Expression

**FIGURE 2:**
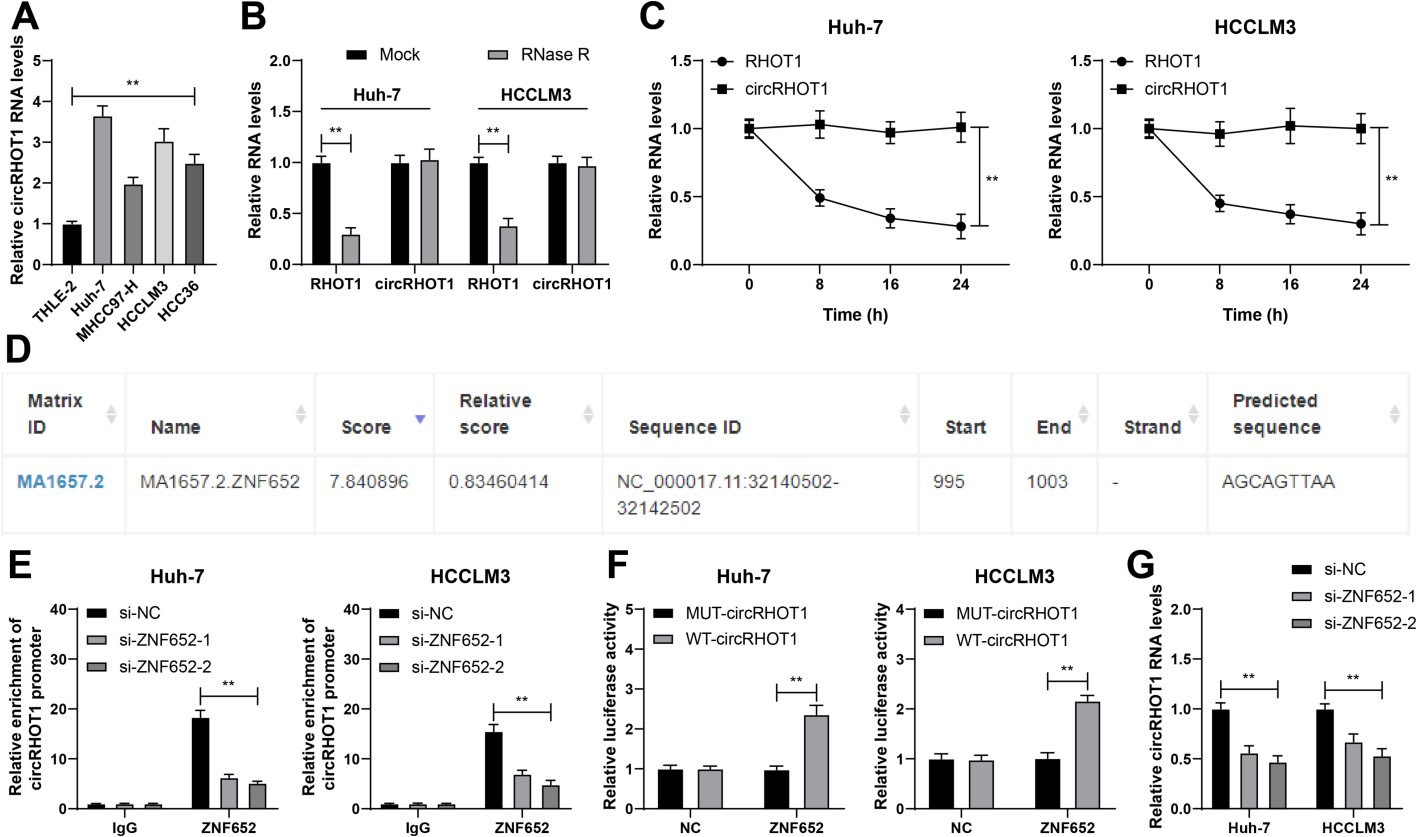
ZNF652 transcriptionally promotes circRHOT1 expression. (A) circRHOT1 expression was assessed via RT–qPCR. (B and C) circRHOT1 stability after RNase R (B) and ACT‐D (C) treatment was detected via RT–qPCR. (D) The binding sites between ZNF652 and the circRHOT1 promoter were predicted through the JASPAR database. (E and F) The binding relationship between ZNF652 and the circRHOT1 promoter was analyzed via ChIP (E) and dual‐luciferase reporter gene assays (F). (G) circRHOT1 expression was assessed via RT–qPCR. Three independent operations were conducted. The data are presented as the means ± standard deviations. One‐way ANOVA was used to compare the data among multiple groups in Panel A, and two‐way ANOVA was used to compare the data among multiple groups in Panels B, C, E–G. Tukey's multiple comparisons test was used for post hoc tests. **p < 0.01.

Herein, it was hypothesized that circRHOT1 could be a downstream molecule of ZNF652 in HCC. RT–qPCR revealed that circRHOT1 was highly expressed in HCC (*p* < 0.01; Figure 2A). circRHOT1 stability in the HCC cell lines was further assessed (*p* < 0.01; Figure 2B,C). The JASPAR database revealed the binding sites between ZNF652 and the circRHOT1 promoter (Figure 2D). ChIP assays revealed that ZNF652 could be enriched in the circRHOT1 promoter region, and the enrichment degree was reduced upon ZNF652 silencing (*p* < 0.01; Figure 2E). Luciferase activity in the HCC cell lines increased upon co‐transfection of pcDNA3.1 ZNF652 and WT‐circRHOT1, further confirming the binding relationship between ZNF652 and circRHOT1 (*p* < 0.01; Figure 2F). CircRHOT1 expression was downregulated upon silencing ZNF652 (*p* < 0.01; Figure 2G). In general, ZNF652 transcriptionally activated circRHOT1 expression.

### 3.3 CircRHOT1 Overexpression Restrains the Role of ZNF652 Silencing in HCC Cell Proliferation and Apoptosis

**FIGURE 3:**
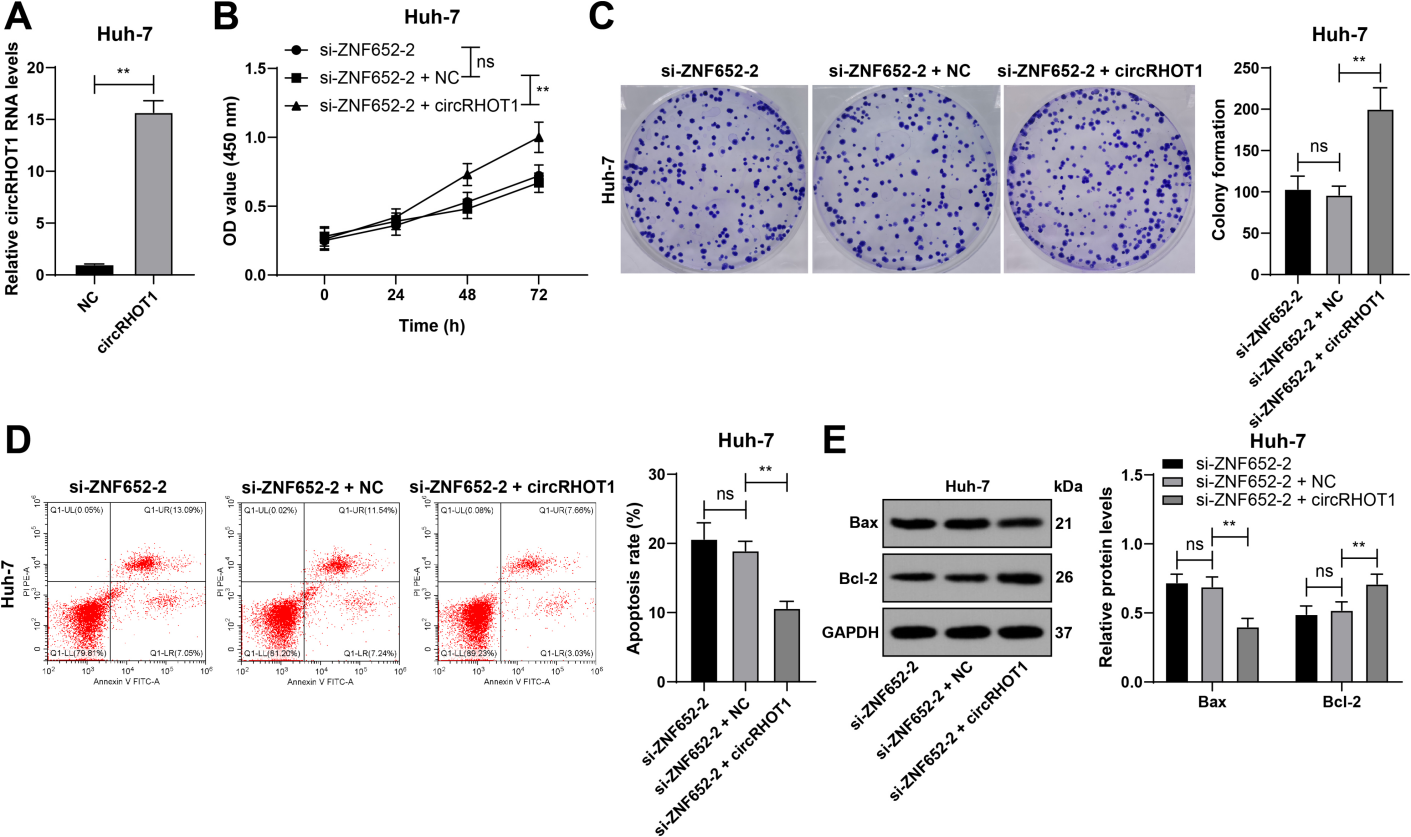
circRHOT1 overexpression restrains the role of ZNF652 silencing in HCC cell proliferation and apoptosis. pcDNA3.1 circRHOT1 was transfected into Huh‐7 cells, with pcDNA3.1 NC as the NC. (A) circRHOT1 transfection efficiency was determined via RT–qPCR. (B) Cell proliferation was verified via the CCK‐8 method. (C) The number of cell colonies was evaluated using a colony formation assay. (D) Apoptosis was assessed via flow cytometry. (E) The expression of Bax and Bcl‐2 was detected via western blot analysis. Three independent operations were conducted. The data are presented as the means ± standard deviations. A *t* test was used to compare the data between two groups in Panel A, one‐way ANOVA was used to compare the data among multiple groups in Panels C and D, and two‐way ANOVA was used to compare the data among multiple groups in Panels B and E. Tukey's multiple comparisons test was used for post hoc tests. ns *p* > 0.05, **p < 0.01.

Combined experiments were conducted in which circRHOT1 was overexpressed and ZNF652 was silenced in Huh‐7 cells (*p* < 0.01; Figure 3A). circRHOT1 increased HCC cell proliferation and colony number (*p* < 0.01; Figure 3B,C). Compared with the si‐ZNF652 group, the si‐ZNF652 + circRHOT1 group showed decreased apoptosis and Bax protein levels and increased Bcl‐2 protein levels (*p* < 0.01; Figure 3D,E), illustrating that circRHOT1 overexpression restrained the interaction of ZNF652 silencing with HCC cell proliferation and apoptosis.

### 3.4 CircRHOT1 Recruits KAT5 to the SLC38A6 Promoter, Inducing H3K27ac Modification to Increase SLC38A6 Expression

**FIGURE 4:**
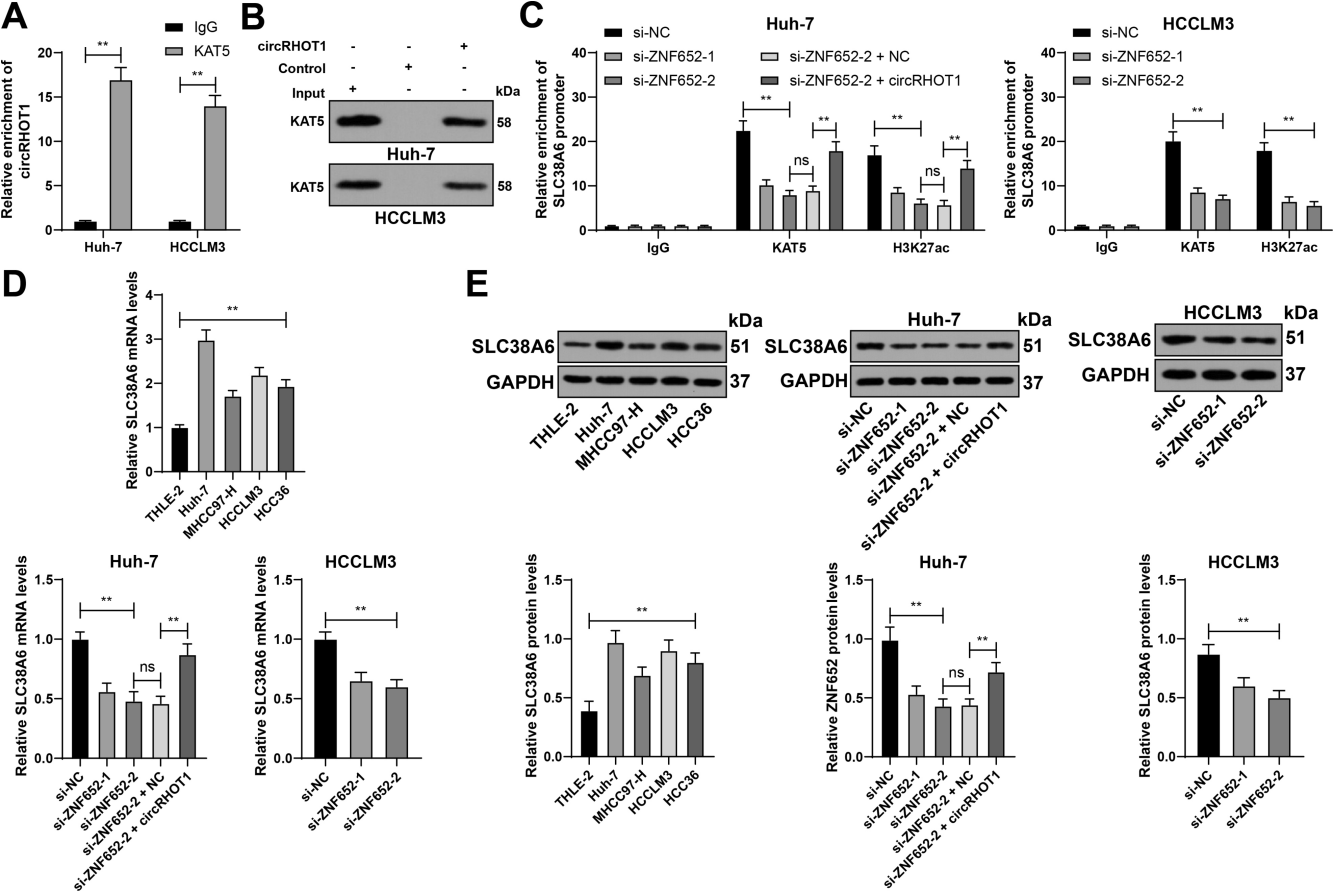
circRHOT1 recruits KAT5 to the SLC38A6 promoter, inducing H3K27ac modification to increase SLC38A6 expression. (A and B) The binding relationship between circRHOT1 and KAT5 was detected via RIP (A) and RNA pull‐down (B) assays. (C) The enrichment of KAT5 and H3K27ac on the SLC38A6 promoter was analyzed via ChIP. (D and E) SLC38A6 expression was determined via RT–qPCR (D) and western blot analysis (E). Three independent experiments were performed. The data are presented as the means ± standard deviations. One‐way ANOVA was used to compare the data among multiple groups in Panels D and E, and two‐way ANOVA was used to compare the data among multiple groups in Panels A and C. Tukey's multiple comparisons test was used for post hoc tests. ns *p* > 0.05, **p < 0.01.

The binding relationship between circRHOT1 and KAT5 in HCC cell lines was validated via RIP and RNA pull‐down assays (*p* < 0.01; Figure 4A,B). ChIP revealed that KAT5 and H3K27ac were recruited to the SLC38A6 promoter (*p* < 0.01; Figure 4C). Enrichment of KAT5 and H3K27ac on the SLC38A6 promoter decreased upon ZNF652 silencing but increased upon circRHOT1 overexpression (*p* < 0.01; Figure 4C). Compared with THLE‐2 cells, HCC cell lines presented elevated expression of SLC38A6 (*p* < 0.01; Figure 4D,E). Compared with the si‐NC group, the si‐ZNF652 group exhibited decreased SLC38A6 expression, whereas compared with the si‐ZNF652‐2 + NC group, the si‐ZNF652‐2 + circRHOT1 group exhibited increased SLC38A6 expression (*p* < 0.01; Figure 4D,E). In conclusion, circRHOT1 recruited KAT5 to the SLC38A6 promoter, inducing H3K27ac modification to increase SLC38A6 expression.

### 3.5 SLC38A6 Overexpression Counteracts the Role of ZNF652 Silencing in HCC Cell Proliferation and Apoptosis

**FIGURE 5:**
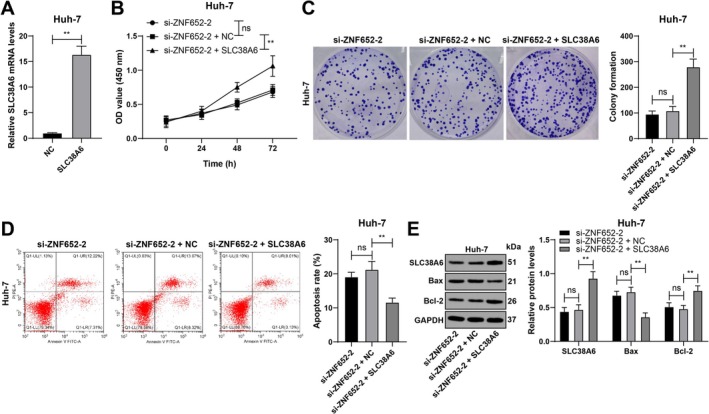
SLC38A6 overexpression counteracts the role of ZNF652 silencing in HCC cell proliferation and apoptosis. pcDNA3.1 SLC38A6 was transfected into Huh‐7 cells, with pcDNA3.1 NC as the NC. (A) SLC38A6 transfection efficiency was evaluated via RT–qPCR. (B) Cell proliferation was verified via the CCK‐8 method. (C) The number of cell colonies was evaluated using a colony formation assay. (D) Apoptosis was assessed via flow cytometry. (E) The expression of SLC38A6, Bax, and Bcl‐2 was detected via western blot analysis. Three independent operations were conducted. The data are presented as the means ± standard deviations. A *t* test was used to compare the data between two groups in Panel A, one‐way ANOVA was used to compare the data among multiple groups in Panels C and D, and two‐way ANOVA was used to compare the data among multiple groups in Panels B and E. Tukey's multiple comparisons test was used for post hoc tests. ns *p* > 0.05, **p < 0.01.

Combined experiments were conducted in which SLC38A6 was overexpressed and ZNF652 was silenced in Huh‐7 cells (*p* < 0.01; Figure 5A,E). SLC38A6 overexpression increased HCC cell viability and colony number (*p* < 0.01; Figure 5B,C), reduced apoptosis, decreased Bax protein expression, and increased Bcl‐2 protein expression (*p* < 0.01; Figure 5D,E).

## 4 Discussion

HCC remains a predominant malignancy worldwide and is associated with an increased likelihood of death, and current treatment protocols are unable to meet therapeutic expectations, resulting in high relapse rates and poor overall survival rates [22]. Mechanistically, the alleviation of HCC is necessarily related to the inhibition of cancer cell proliferation and the activation of apoptosis [23]. Our experiment was designed to identify possible treatments for HCC through the exploration of ZNF652 and its potential downstream mechanism. We found that ZNF652 transcriptionally activated circRHOT1 expression, recruited KAT5 to the SLC38A6 promoter, enhanced H3K27ac enrichment, and activated SLC38A6 expression, thus promoting HCC cell proliferation and inhibiting apoptosis.

Initially, we reported that ZNF652 expression was upregulated in HCC. Similarly, Li et al. reported that ZNF652 is abundantly expressed in patients with HCC and that it stimulates cancer cell pathogenesis, aggressiveness, and metabolism [24]. However, the concrete effect of ZNF652 on HCC progression should be extensively discussed. Here, ZNF652 was silenced in HCC cell lines, after which proliferation, viability, and colony number decreased and apoptosis increased, as indicated by elevated Bax protein expression and suppressed Bcl‐2 protein expression. ZNF521 can abrogate cancer apoptosis while promoting cell growth and viability in gastric cancer [25]. Similarly, ZNF224 plays an oncogenic role in breast cancer by activating cancer cell development and inhibiting apoptosis [26], indicating that ZNF proteins might be candidate targets for cancer apoptosis. ZNF652 expression is activated in gastric cancer, which accelerates cell proliferation and strengthens drug resistance to therapies [27], suggesting the oncogenic feature of ZNF652. ZNF652 can influence malignant cell proliferation, metabolism, and apoptosis by interacting with its downstream targets to manipulate gene expression and biological behaviors in cancers [28]. Therefore, we explored the downstream mechanisms mediated by ZNF652.

ZNF652 modulates the expression and transcription of various genes to influence malignant molecular processes by transcriptionally mediating the expression of its downstream genes [29, 30]. Among the targets binding to ZNF652, circRHOT1 was positively associated with ZNF652 to impair bladder cancer treatment [12], which is consistent with our finding that ZNF652 could transcriptionally activate circRHOT1 expression. Similarly, the binding relationships between circRHOT1 and other RNAs or transcription factors are highly important for cancer growth and migration [31, 32], further supporting our hypothesis that ZNF652 activates circRHOT1 expression to exacerbate HCC malignancy. In our study, circRHOT1 expression was upregulated, after which HCC cell proliferation, viability, and colony number increased, apoptosis decreased, Bax expression decreased, and Bcl‐2 expression increased. CircRHOT1 is robustly expressed in HCC and can enhance HCC proliferation and mobility, leading to a disappointing overall survival rate [13]. The overexpression of circRHOT1 in breast cancer promotes cancer cell activity, mobility, and metabolism and slows apoptosis [33]. Next, the downstream mechanism of circRHOT1 was elucidated. CircRHOT1 accumulates KAT5 to increase glutathione peroxidase‐4 H3K27ac enrichment and facilitate its transcription, thereby exacerbating gastric cancer malignancy and impeding malignant cell death and apoptosis [34]. KAT5 is strongly expressed in HCC, and it can strengthen the oncogenic effect of its upstream molecules to further stimulate tumor growth [35]. In addition, when KAT5 is recruited by a related gene, H3K27ac enrichment is correspondingly increased [36]. H3K27ac enrichment has been detected in the SLC38A6 promoter. SLC38A6 is upregulated in HCC and results in a poor prognosis and a short recurrence‐free period [37]. In conclusion, circRHOT1 recruits KAT5 to the SLC38A6 promoter, inducing H3K27ac modification to increase SLC38A6 expression. With respect to the role of SLC38A6 in HCC, SLC38A6 was shown to be upregulated, and HCC cell proliferation, viability, and colony number were increased, apoptosis was decreased, Bax expression was decreased, and Bcl‐2 expression was increased. SLC38A6 is highly related to the pathogenesis, development, progression, and drug resistance of a variety of carcinomas [38, 39]. On the other hand, SLC38A6 depletion is beneficial for alleviating cancer, as it limits cell proliferation and promotes apoptosis [40]. Overall, SLC38A6 overexpression accelerated HCC cell proliferation and inhibited apoptosis.

This study has several limitations. We investigated this mechanism only at the cellular level. We acknowledge that the lack of in vivo studies and patient sample validation is a limitation of our study. In addition, the upstream mechanism of ZNF652 should also be investigated to further confirm our results, and the possible competing endogenous RNA network should also be explored. In future research, we will further explore the possible upstream mechanism of ZNF652 to promote the search for HCC treatment.

## 5 Conclusions

In summary, our results supported that ZNF652 transcriptionally activated circRHOT1 expression, recruited KAT5 to the SLC38A6 promoter, increased H3K27ac enrichment, and upregulated SLC38A6 expression, thus promoting HCC cell proliferation and inhibiting apoptosis. These results are promising for promoting HCC therapy involving ZNF652.

## Conflicts of Interest

The authors declare no conflicts of interest.

## Data Availability

The data that support the findings of this study are available from the corresponding author upon reasonable request.
